# Supplementary material for: Environmental changes in oxygen tension reveal ROS-dependent neurogenesis and regeneration in the adult newt brain
Source: eLife. 2015 Oct 20;4:e08422. doi: 10.7554/eLife.08422 (PMC4635398; doi:10.7554/eLife.08422)
Supplement: Figure 1—source data 1. — DOI: http://dx.doi.org/10.7554/eLife.08422.004 [file elife08422s001.docx]

**Table 1: Figure 1B (Number of TUNEL+)**

| Forebrain | Control | Hypoxia | Re-oxygenation |
| --- | --- | --- | --- |
| 1 | 30 | 60 | 70 |
| 2 | 25 | 35 | 75 |
| 3 | 20 | 50 | 100 |
| 4 | 25 | 50 | 80 |

**Table 2: Figure 1C (Number of NeuN+/TUNEL+)**

| Forebrain | Control | Hypoxia | Re-oxygenation |
| --- | --- | --- | --- |
| 1 | 10 | 15 | 20 |
| 2 | 5 | 10 | 25 |
| 3 | 5 | 15 | 15 |
| 4 | 5 | 10 | 20 |

**Table 3: Figure 1D (Number of PCNA+ IBA1+/IBA1+)**

| Forebrain | Control  PCNA+IBA1+/IBA1+ | Hypoxia  PCNA+IBA1+/IBA1+ | Re-oxygenation  PCNA+IBA1+/IBA1+ |
| --- | --- | --- | --- |
| 1 | (35/675) | (5/440) | (305/1425) |
| 2 | (15/1710) | (325/1920) | (290/2040) |
| 3 | (100/1525) | (145/2985) | (1185/6685) |
| 4 | (100/1610) | (230/2150) | (85/1500) |
| 5 | (175/2250) |  |  |
