# Supplementary material for: Environmental changes in oxygen tension reveal ROS-dependent neurogenesis and regeneration in the adult newt brain
Source: eLife. 2015 Oct 20;4:e08422. doi: 10.7554/eLife.08422 (PMC4635398; doi:10.7554/eLife.08422)
Supplement: Figure 2—source data 1. — DOI: http://dx.doi.org/10.7554/eLife.08422.007 [file elife08422s002.docx]

**Table 1: Figure 2 - Figure supplement 1B (Number of EdU+ GFAP+ cells)**

| Forebrain | Control  EdU+GFAP+ | Reoxygenation  EdU+GFAP+ |
| --- | --- | --- |
| 1 | 130 | 235 |
| 2 | 95 | 180 |
| 3 | 170 | 275 |
| 4 | 155 | 320 |
