# Supplementary material for: Environmental changes in oxygen tension reveal ROS-dependent neurogenesis and regeneration in the adult newt brain
Source: eLife. 2015 Oct 20;4:e08422. doi: 10.7554/eLife.08422 (PMC4635398; doi:10.7554/eLife.08422)
Supplement: Figure 2—source data 2. — DOI: http://dx.doi.org/10.7554/eLife.08422.008 [file elife08422s003.docx]

**Table 1: Figure 2B (Number of PCNA+ GFAP+ cells)**

| Forebrain | Control  PCNA+GFAP+ | Hypoxia  PCNA+GFAP+ |
| --- | --- | --- |
| 1 | 1045 | 790 |
| 2 | 465 | 650 |
| 3 | 555 | 665 |
| 4 | 970 | 525 |

**Table 2: Figure 2C (Number of PCNA+ GFAP+ cells)**

| Forebrain | Control  PCNA+GFAP+ | Reoxygenation  PCNA+GFAP+ |
| --- | --- | --- |
| 1 | 370 | 825 |
| 2 | 330 | 850 |
| 3 | 575 | 375 |
| 4 | 365 | 740 |
| 5 | 385 | 885 |

**Table 3: Figure 2E (Number of Hu+ EdU+ cells)**

| Forebrain | Control  Hu+ EdU+ | Reoxygenation  Hu+ EdU+ |
| --- | --- | --- |
| 1 | 10 | 235 |
| 2 | 120 | 280 |
| 3 | 105 | 110 |
| 4 | 80 | 165 |
| 5 | 110 | 220 |
