# Supplementary material for: Environmental changes in oxygen tension reveal ROS-dependent neurogenesis and regeneration in the adult newt brain
Source: eLife. 2015 Oct 20;4:e08422. doi: 10.7554/eLife.08422 (PMC4635398; doi:10.7554/eLife.08422)
Supplement: Figure 3—source data 1. — DOI: http://dx.doi.org/10.7554/eLife.08422.011 [file elife08422s004.docx]

**Table 1: Figure 3 - Figure supplement 1 B (Mean intensity of HEt^+^/DCX^+^ and HEt^+^/GFAP^+^)**

| Sample 1  DCX/HEt | Sample 2  DCX/HEt | Sample 3  DCX/HEt | Sample 1  GFAP/HEt | Sample 2  GFAP/HEt | Sample 3  GFAP/HEt |
| --- | --- | --- | --- | --- | --- |
| 55,965 | 39,527 | 60,91 | 25,95 | 43,422 | 114,997 |
| 30,706 | 47,677 | 115,569 | 21,728 | 46,657 | 76,788 |
| 31,396 | 88,201 | 66,743 | 20,009 | 28,851 | 78,752 |
| 31,767 | 89,527 | 93,398 | 16,26 | 38,237 | 52,223 |
| 39,915 | 59,586 | 46,982 | 14,543 | 35,126 | 70,986 |
| 20,389 | 34,287 | 88,883 | 18,989 | 31,373 | 80,142 |
| 39,772 | 34,249 | 30,082 | 40,787 | 72,27 | 50,013 |
| 51,607 | 67,5 | 43,087 | 24,551 | 89,834 | 64,425 |
| 39,934 | 49,157 | 33,124 | 51,418 | 53,082 | 47,797 |
| 41,623 | 36,839 | 71,471 | 36,806 | 89,378 | 61,399 |
| 38,346 | 75,248 | 79,52 | 15,235 | 22,965 | 88,942 |
| 46,448 | 52,704 | 41,934 |  | 15,743 | 120,208 |
| 27,505 | 52,889 |  |  |  | 140,992 |
| 18,654 | 96,920 |  |  |  | 96,613 |
|  | 109,024 |  |  |  | 145,552 |
|  | 84,397 |  |  |  | 81,866 |
|  |  |  |  |  | 57,548 |
|  |  |  |  |  | 61,427 |
|  |  |  |  |  | 28,248 |
|  |  |  |  |  | 60,841 |
|  |  |  |  |  | 65,416 |
|  |  |  |  |  | 76,201 |
|  |  |  |  |  | 41,394 |
|  |  |  |  |  | 24,065 |
|  |  |  |  |  | 29,555 |
|  |  |  |  |  | 48 |
|  |  |  |  |  | 51,896 |
|  |  |  |  |  | 37,03 |
|  |  |  |  |  | 49,332 |
|  |  |  |  |  | 45,049 |
|  |  |  |  |  | 55,513 |
