# Supplementary material for: Environmental changes in oxygen tension reveal ROS-dependent neurogenesis and regeneration in the adult newt brain
Source: eLife. 2015 Oct 20;4:e08422. doi: 10.7554/eLife.08422 (PMC4635398; doi:10.7554/eLife.08422)
Supplement: Figure 3—source data 2. — DOI: http://dx.doi.org/10.7554/eLife.08422.012 [file elife08422s005.docx]

**Table 1: Figure 3B (Number of PCNA+ GFAP+ cells)**

| Forebrain | Control  PCNA+GFAP+ | Control/Apocynin  PCNA+GFAP+ |
| --- | --- | --- |
| 1 | 350 | 365 |
| 2 | 500 | 430 |
| 3 | 335 | 385 |
| 4 | 540 | 305 |

**Table 2: Figure 3D (Number of PCNA+ GFAP+ cells)**

| Forebrain | Reoxygenation  PCNA+GFAP+ | Reoxygenation/Apocynin  PCNA+GFAP+ |
| --- | --- | --- |
| 1 | 935 | 450 |
| 2 | 605 | 195 |
| 3 | 740 | 390 |
| 4 | 1230 | 715 |

**Table 3: Figure 3 F (Number of PCNA+ GFAP+ cells)**

| Forebrain | Reoxygenation  PCNA+GFAP+ | Reoxygenation/Mitotempo  PCNA+GFAP+ |
| --- | --- | --- |
| 1 | 355 | 740 |
| 2 | 420 | 395 |
| 3 | 485 | 350 |
| 4 | 430 | 480 |

**Table 4: Figure 3H (Number of Hu+ EdU+ cells)**

| Forebrain | Reoxygenation  Hu+ EdU+ | Reoxygenation/Apocynin  Hu+ EdU+ |
| --- | --- | --- |
| 1 | 235 | 145 |
| 2 | 280 | 110 |
| 3 | 220 | 95 |
| 4 | 165 | 125 |
| 5 | 110 |  |
