# Supplementary material for: Environmental changes in oxygen tension reveal ROS-dependent neurogenesis and regeneration in the adult newt brain
Source: eLife. 2015 Oct 20;4:e08422. doi: 10.7554/eLife.08422 (PMC4635398; doi:10.7554/eLife.08422)
Supplement: Figure 4—source data 1. — DOI: http://dx.doi.org/10.7554/eLife.08422.015 [file elife08422s006.docx]

**Table 1: Figure 4 - Figure supplement 1 A (Number of PCNA+ IBA1+/IBA1+ cells)**

| Forebrain | Re-oxygenation | Re-oxygenation/Apocynin |
| --- | --- | --- |
| 1 | (405/1775) | (280/1025) |
| 2 | (260/1155) | (610/2740) |
| 3 | (30/85) | (555/2460) |
| 4 | (2600/5610) | (80/220) |

**Table 2: Figure 4 - Figure supplement 1 B (Number of Neurosphere number)**

| Forebrain | Control | Control /Apocynin |
| --- | --- | --- |
| 1 | 12 | 14 |
| 2 | 15 | 16 |
| 3 | 14 | 10 |
| 4 | 18 | 12 |

**Table 3: Figure 4 - Figure supplement 1 C (Number of Neurosphere number)**

| Neural stem cells | Control | Re-oxygenation | Re-oxygenation/Apocynin |
| --- | --- | --- | --- |
| 1 | 10 | 18 | 14 |
| 2 | 18 | 32 | 18 |
| 3 | 15 | 24 | 22 |
| 4 | 20 | 32 | 19 |
| 5 | 13 | 23 | 11 |
| 6 | 15 | 18 | 13 |
