# Supplementary material for: Environmental changes in oxygen tension reveal ROS-dependent neurogenesis and regeneration in the adult newt brain
Source: eLife. 2015 Oct 20;4:e08422. doi: 10.7554/eLife.08422 (PMC4635398; doi:10.7554/eLife.08422)
Supplement: Figure 4—source data 2. — DOI: http://dx.doi.org/10.7554/eLife.08422.016 [file elife08422s007.docx]

**Table 1: Figure 4 B (Number of PCNA+ IBA1+/IBA1+)**

| **Forebrain** | **Re-oxygenation** | **Re-oxygenation/Dexamethasone** |
| --- | --- | --- |
| **1** | (2080/5750) | (305/1525) |
| **2** | (340/1575) | (225/1110) |
| **3** | (630/2500) | (315/1935) |
| **4** | (485/1045) | (190/1180) |
| **5** | (1645/3880) | (255/1265) |
| **6** | (850/1360) | (300/1115) |

**Table 2: Figure 4 C (Number of PCNA+ GFAP+)**

| **Forebrain** | **Re-oxygenation** | **Re-oxygenation/Dexamethasone** |
| --- | --- | --- |
| **1** | 660 | 775 |
| **2** | 545 | 645 |
| **3** | 515 | 530 |
| **4** | 475 | 430 |
| **5** | 455 | 280 |
| **6** | 920 | 805 |

**Table 3: Figure 4 F (Number of EdU+ GFAP+/GFAP+)**

| Neural stem cells | Control | Re-oxygenation | Re-oxygenation/Apocynin |
| --- | --- | --- | --- |
| 1 | (181/319) | (82/104) | (97/171) |
| 2 | (56/112) | (87/129) | (36/67) |
| 3 | (39/60) | (168/244) | (45/75) |
|  |  |  |  |

**Table 4: Figure 4 G (Number of EdU+ GFAP+/GFAP+)**

| Neural stem cells | Control | Control/Apocynin |
| --- | --- | --- |
| 1 | (181/319) | (31/67) |
| 2 | (56/112) | (29/62) |
| 3 | (39/60) | (94/178) |
