# Supplementary material for: Environmental changes in oxygen tension reveal ROS-dependent neurogenesis and regeneration in the adult newt brain
Source: eLife. 2015 Oct 20;4:e08422. doi: 10.7554/eLife.08422 (PMC4635398; doi:10.7554/eLife.08422)
Supplement: Figure 5—source data 1. — DOI: http://dx.doi.org/10.7554/eLife.08422.019 [file elife08422s008.docx]

**Table 1: Figure 5 C (Number of PCNA+ GFAP+)**

| Midbrain | 6-OHDA | 6-OHDA/Apocynin |
| --- | --- | --- |
| 1 | 190 | 50 |
| 2 | 190 | 50 |
| 3 | 285 | 75 |
| 4 | 85 | 55 |

**Table 2: Figure 5 E (Number of TH+)**

| Midbrain | 6-OHDA | 6-OHDA/Apocynin |
| --- | --- | --- |
| 1 | 160 | 30 |
| 2 | 210 | 25 |
| 3 | 55 | 60 |
| 4 | 170 | 30 |
| 5 | 50 |  |

**Table 3: Figure 5 F (Number of TH+)**

| Midbrain | Sham | Sham/Apocynin |
| --- | --- | --- |
| 1 | 265 | 235 |
| 2 | 230 | 345 |
| 3 | 270 | 290 |
| 4 | 325 | 270 |
